# Supplementary material for: Causal Relationship between Aging and Anorexia Nervosa: A White-Matter-Microstructure-Mediated Mendelian Randomization Analysis
Source: Biomedicines. 2024 Aug 16;12(8):1874. doi: 10.3390/biomedicines12081874 (PMC11351342; doi:10.3390/biomedicines12081874)
Supplement: Supplementary file 1 [file biomedicines-12-01874-s001.zip › Supplementary Figure/Figure S4/Singe SNP plot of MR analysis for TL to ubm-b-1798.pdf]

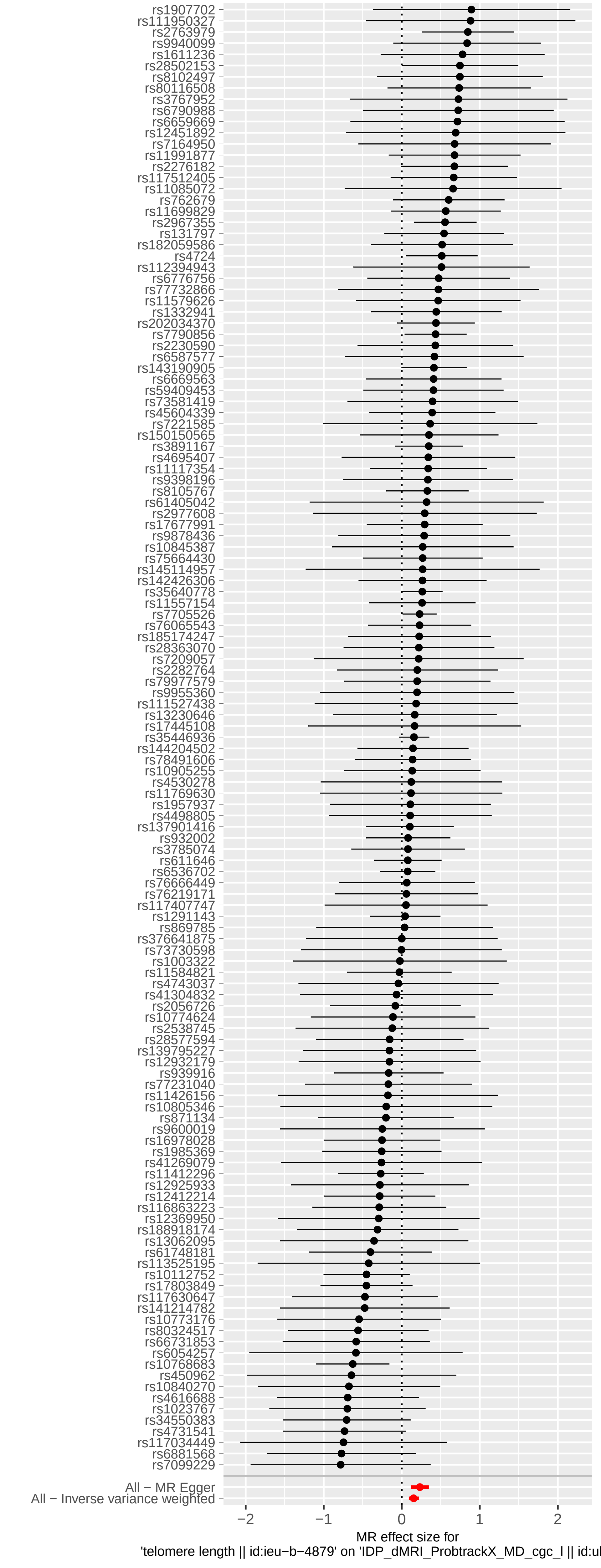
rs1907702
rs111950327
rs2763979
rs9940099
rs1611236
rs28502153
rs8102497
rs80116508
rs3767952
rs6790988
rs6659669
rs12451892
rs7164950
rs11991877
rs2276182
rs117512405
rs11085072
rs762679
rs11699829
rs2967355
rs131797
rs182059586
rs4724
rs112394943
rs6776756
rs77732866
rs11579626
rs1332941
rs202034370
rs7790856
rs2230590
rs6587577
rs143190905
rs6669563
rs59409453
rs73581419
rs45604339
rs7221585
rs150150565
rs3891167
rs4695407
rs11117354
rs9398196
rs8105767
rs61405042
rs2977608
rs17677991
rs9878436
rs10845387
rs75664430
rs145114957
rs142426306
rs35640778
rs11557154
rs7705526
rs76065543
rs185174247
rs28363070
rs7209057
rs2282764
rs79977579
rs9955360
rs111527438
rs13230646
rs17445108
rs35446936
rs144204502
rs78491606
rs10905255
rs4530278
rs11769630
rs1957937
rs4498805
rs137901416
rs932002
rs3785074
rs611646
rs6536702
rs76666449
rs76219171
rs117407747
rs1291143
rs869785
rs376641875
rs73730598
rs1003322
rs11584821
rs4743037
rs41304832
rs2056726
rs10774624
rs2538745
rs28577594
rs139795227
rs12932179
rs939916
rs77231040
rs11426156
rs10805346
rs871134
rs9600019
rs16978028
rs1985369
rs41269079
rs11412296
rs12925933
rs12412214
rs116863223
rs12369950
rs188918174
rs13062095
rs61748181
rs113525195
rs10112752
rs17803849
rs117630647
rs141214782
rs10773176
rs80324517
rs66731853
rs6054257
rs10768683
rs450962
rs10840270
rs4616688
rs1023767
rs34550383
rs4731541
rs117034449
rs6881568
rs7099229
All − MR Egger
All − Inverse variance weighted
−2
−1
0
1
2
MR effect size for
'telomere length || id:ieu−b−4879' on 'IDP_dMRI_ProbtrackX_MD_cgc_l || id:ul
